# Endoscopic Dacryocystorhinostomy (DCR): a comparative study between powered and non-powered technique

**DOI:** 10.1186/s40463-015-0109-z

**Published:** 2015-12-22

**Authors:** Islam Herzallah, Bassam Alzuraiqi, Naif Bawazeer, Osama Marglani, Ameen Alherabi, Sherif K. Mohamed, Khalid Al-Qahtani, Talal Al-Khatib, Abdullah Alghamdi

**Affiliations:** Department of Otolaryngology, Zagazig University, Zagazig, Egypt; Department of Otolaryngology-Head & Neck Surgery, King Abdullah Medical City, Makkah, Saudi Arabia; Department of Otolaryngology-Head & Neck Surgery, Umm Al-Qura University, Makkah, Saudi Arabia; Department of Otolaryngology, Ain Shams University, Cairo, Egypt; Department of Otolaryngology-Head and Neck Surgery, King Saud University, Riyadh, Saudi Arabia; Department of Otolaryngology-Head & Neck Surgery, King Abdulaziz University, Jeddah, Saudi Arabia; Department of Ophthalmology, Umm Al-Qura University, Makkah, Saudi Arabia; P.O.Box 41405, Jeddah, 21521 Saudi Arabia

**Keywords:** Kerrison, Drill, Endoscopic Dacryocystorhinostomy, DCR

## Abstract

**Background:**

Dacrocystorhinostomy (DCR) is an operation used to treat nasolacrimal duct obstruction. Essentially there are two approaches: external and endoscopic. Several modalities are used in endoscopic DCR; all aiming to improve success rate, reduce complications, and shorten operative time. Both kerrison punch and drill are widely used in endoscopic DCR with non-conclusive knowledge about differences in operative details as well as on the outcome. The aim of this study is to compare between powered (drill) and non-powered (kerrison punch) DCR to clarify the superiority of one over the other.

**Methods:**

A retrospective chart review of 59 patients who underwent endoscopic DCR procedure at our institution from June 2013 until July 2014 (34 kerrison punch and 32 powered drill). Operative details, surgical outcome and complications were compared between both groups.

**Results:**

A total of 66 endoscopic DCRs were performed on 59 patients. Procedure success rate among kerrison punch group was 87.88 % vs. 90.9 % in powered drill group (*p* = 0.827), while complications for both groups were statistical not significant (*p* = 0.91). The mean operating time among kerrison punch group was significantly lower than in powered drill group (75 min vs. 125 min, *p* = 0.0001).

**Conclusion:**

Kerrison punch showed significant reduction in operating time when compared to powered drill for endoscopic DCR. No statistically significant difference was found between both groups regarding procedures’ success rate and complication.

## Background

Epiphora due to nasolacrimal duct obstruction is a common clinical problem that can be caused by functional or anatomical abnormality. An anatomical obstruction could be at any point along the lacrimal excretory system and could be congenital or acquired. The primary acquired nasolacrimal duct obstruction is believed to occur due to chronic inflammatory process resulting in fibrosis, stenosis, and closure of the duct ostium [1]. This can be managed surgically by dacryocystorhinostomy (DCR), which is used to create a fistula that bypasses the obstruction and restores the tear flow. The operative approach could be external or an endoscopic approach. External DCR was the gold standard method even after the endoscopic approach had been described, because of limited technology at that time with a success rate ranging between 80 and 100 % [2]. However, improvements of visualization & instrumentation technology made the endoscopic DCR gain its popularity. In addition, endoscopic DCR has several advantages over external DCR including: no external incision, shorter recovery time, maintenance of the lacrimal pumping mechanism and lower postoperative morbidity [3].

Several modalities and adjuncts such as Kerrison punch, powered drill, and lasers have been described in endoscopic DCR with the aim of improving operative technique and success rate [2]. However, the variety of tools used in endoscopic DCR made it difficult to determine the best approach, and thus a comparison between some of the available techniques seems to be important to know the advantages and disadvantages. Both kerrison punch and powered drill are widely used in endoscopic DCR with slowly expanding knowledge about the differences in operative details as well as in the surgical outcome [4]. Our objective is to compare those two modalities and try to clarify the superiority of one technique over the other.

## Methods

### Study design

A retrospective review was conducted after creation and development of an electronic DCR DATABASE using Microsoft^©^ Access 2012 (Microsoft Corporation) on all patients underwent endoscopic DCR procedure at our institution from July 2013 until June 2014. Appropriate patients’ demographics, diagnoses, radiological evaluation, surgical details, complications, and outcomes were included. Exclusion criteria were posttraumatic lacrimal obstruction, congenital cases, cases with combined other sino-nasal procedures (e.g. septoplasty, turbinate procedures, sinus surgery), cases were both drill & kerisson used together, and cases with follow up less than three months. Institutional review board approval of the study protocol was obtained prior to initiation of the study.

### Surgical technique

All patients received a standard preoperative assessment including history & physical examination, endoscopic evaluation, and CT scan of paranasal sinuses to exclude possible sinonasal pathologies. Diagnosis of nasolacrimal duct obstruction was made by classical symptomatic presentation along with fluorescein dye test, and syringing test.

**Fig. 1:**
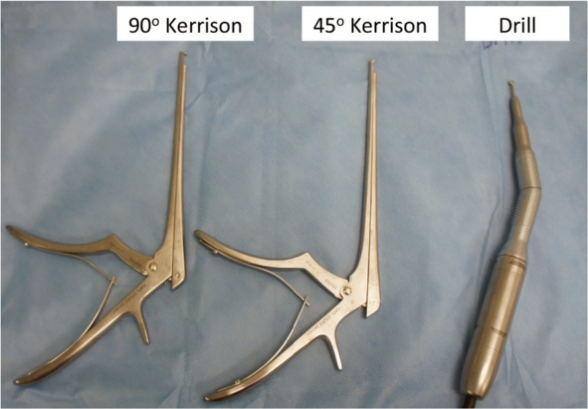
Instruments compared in this study; namely 90°, 45° kerrison punch, and powered drill

**Fig. 2:**
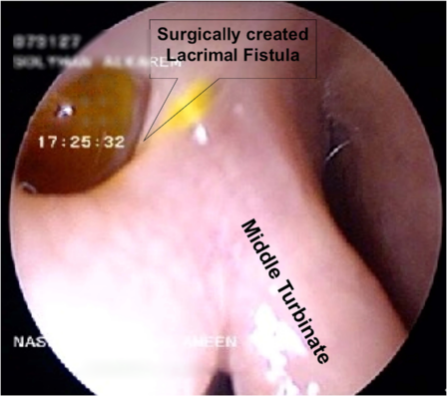
Three months post operative endoscopic examination showing positive fluorescein test and patent lacrimal fistula

All operations were performed under general anesthesia. Endoscopic DCR was done using a standard surgical technique. For the Osteotomy part of the surgery, two different instruments were used to remove the bone of the maxillary frontal process. In the first group, the powered drill was used; while in the second group the kerisson punch was utilized to get sufficient exposure of the lacrimal sac, see Fig. 1. Once the drill or the kerisson was used, no instrument conversion was allowed. Standard silicone stent was used to stent the lacrimal canaliculi. Operating time represents the duration from lateral nasal mucosal incision till the stent is secured. All surgeries were performed; or under direct supervision, of the two senior surgeons with comparable experience and training (O.M & A.A). Postoperatively, outpatient standardized follow up were scheduled at one week then 1, 3, 6, & 12 months. Further follow up was individualized as per patient cases especially those requiring the other side to be done. Three criteria were used to judge success of the operation: the patient expressed improvement of the epiphora, a positive fluorescein test, and patent fistula during endoscopic examination [5] see Fig. 2.

Data were analyzed using SPSS software® (version 16). The Independent two-sample *t*-test was used to assess significance between variables and a value of *P* < 0.05 was taken as statistically significant with a confidence interval level of 95 %.

## Results

**Fig. 3:**
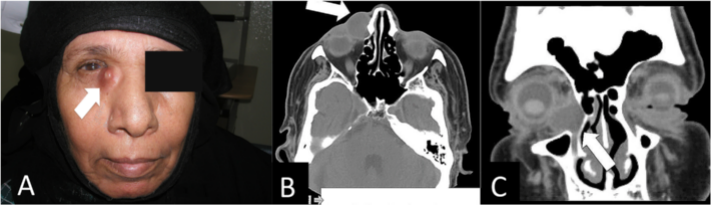
Preoperative diagnosis. **a** A Clinical photo of a patient with right eye dacrocele. **b** CT Axial cut showing medial canthus dacrocele. **c** CT Coronal cut showing the same finding

**Fig. 4:**
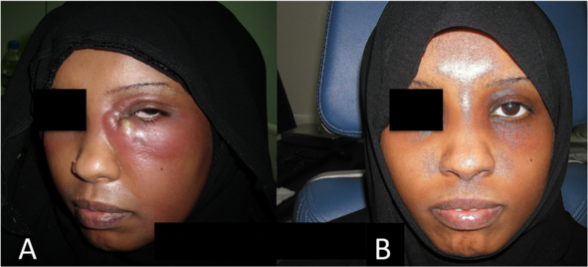
Preoperative diagnosis. **a** A Clinical photo of a patient with left eye acute dacrocystitis. **b** Same patient after medical treatment

A total of 66 endoscopic DCRs were performed on 59 patients. Forty patients were women and 19 were men, with a mean age of 45 years (range 13–96 years). All 59 patients were local residents without any significant racial difference. Thirty-one cases were done in the right eye and 35 in the left eye. The original diagnosis of primary DCRs (59 cases) was acute, recurrent acute, chronic dacryocystitis, and dacroceles in 44 cases; and functional idiopathic epiphora in 15 patients. All patients failed syringing and fluorescein testing. No surgery was done during the acute phase. See Fig. 3 for an example of a case of dacrocele and Fig. 4 for a case if acute dacrocystitis. Eight cases were revision DCR; of which 4 cases were in each group. Thirty-two cases were done using powered drill technique and 34 utilizing kerrison punch. Postoperative follow-up had a mean duration of 8.2 (range = 3–24) months both groups. The mean time for stent removal was 9.5 weeks for both groups; see Table 1 for complete patients demographics.

**Table 1:** Patients demographics & descriptive statistics

		Overall		Instrument used			
				Drill		Kerrison punch	
Age	Range	13–96 Years		13–96 Years		16–75 Years	
	Mean	45		48		41	
Gender	Male	19	32.20 %	11	34.40 %	8	29.60 %
	Female	40	67.80 %	21	65.60 %	19	70.40 %
Eye affected	Right	31	47 %	17	53.13 %	14	41.20 %
	Left	35	53 %	15	46.87 %	20	58.80 %
Stent removal	Range	0–48 Weeks		0–48 weeks		1–20 Weeks	
	Mean	9.5		12		8	
Follow up	Range	3–24 Months		3–24 Months		3–19 Months	
	Mean	8.2		7.03		9.93	

The overall success rate was 89.39 %. The success rate for powered drill group was 90.91 %, compared with 87.88 % for kerrison punch group. The mean operating time of surgery in the powered drill group was 125 min compared to 75 min in the kerrison punch group; see Table 2.

**Table 2:** Procedure success and operating time according to the equipment of endoscopic DCR

		Overall	Powered drill	Kerrison punch	*p* value
			(*n* = 32)	(*n* = 34)	
Success rate		89.39 %	90.91 %	87.88 %	0.827
Operating time (Minutes)	Range	24–210	24–210	30–125	0.0001
	Mean	99.75	125	75	

Reported intraoperative and postoperative complications were all minor and included: intranasal synechiae in two cases, stent accidental fall out in five cases and eye/cheek bruise in three cases, and nostril burn in three cases. The overall minor complication rate was 18 % and there was no record of any major complications. Comparing the two groups it was not statistically significant (*p* = 0.53). Looking into nostril burn alone was also not statistically significant but showed a trend (*p* = 0.1); see Table 3.

**Table 3:** Minor complications of endoscopic DCR

	Drill		Kerrison punch		
	(*n* = 32)		(*n* = 34)		
	n	%	n	%	
Intranasal synechiae	1	1.5	1	1.5	*P* = 0.53
Stent fell out	2	3	3	4.5	
Eye/Cheek bruise	2	3	1	1.5	
Nostril burn	2	3	0	0	
Total	7	10.5	5	7.5	

## Discussion

External DCR was considered superior procedure compared to the endoscopic approach classically, but in the last years there were significant improvements in the technique of endoscopic DCR [3, 6]. These improvements are the result of evolution in surgical instruments, improvement in endoscopic equipment and growing surgical experience [3].

Osteotomy and creation of the bony lacrimal window is a crucial step during endoscopic DCR; a previous study reported that sometimes only 2 % of the original stoma created intra-operatively will remain patent after healing process; but found no statistically valid correlation between the size of the bony opening and the final size of the healed intranasal ostium [7]. Creation of a large bony stoma does not mean successful procedure since minimization of intra-operative tissue damage and postoperative scarring is another key point for success [7, 8]. Other literature, however, showed a relationship between the size of the bony ostium created during DCR surgery and the outcome of the procedure [2, 4, 6, 9]. The creation of the bony window can be achieved by many technical variations including powered drill, kerrison bone punch, radio-surgical electrodes, and lasers. Each instrument has been well described in literature with different results and consequences, but comparison between those instruments and surgical outcome is still non conclusive.

The value of non-traumatic procedure is an emerging concept in endoscopic DCR. The main idea of this concept is to avoid using instruments and tools that might increases the tissue trauma within the surgical field [10]. Trauma could be in form of excessive mechanical force as when using powered drill or can be transmitted heats from cautery and laser assisted instruments. While using powered drill, temperature could reach up to 70 °C at the tip during drilling with possibility of causing local edema and tissue reaction in the postoperative period [11, 12]. Avoiding trauma in this narrow anatomical site will increase chances of first-intention healing process with less formation of scarring and granulation tissue, which ultimately may reduce risk of closure of previously surgically opened lacrimal sac and soft tissue window [4]. In addition, presence of the drill’s rotating shaft within narrow surgical corridor may add some risk to damage nearby tissue [3]. Other disadvantages of powered drills or other high techniques instruments include the possibility of damage to orbital wall or lamina papyracea leading to orbital fat prolapse or penetration to the ethmoidal sinus or skull base with CSF leakage [11]. Nevertheless, with favorable result of non-traumatic endoscopic DCR in theory, published results in the literature showed comparable outcome for drill and punch endoscopic DCR [13–18].

The use of advanced tools like drills is not necessary to increase the success rate for endoscopic DCR in general [8]. Our current study showed similar result, where procedure success rate among kerrison punch group was 87.88 % vs. 90.91 % in the powered drill group (*p* = 0.82); see Table 4 for a summary of some previous studies success rates.

**Table 4:** Comparison between success rates of our study and previous similar studies

Author	Country	Instrument used	Success rate (%)
Ben Simon, et al. [2]	USA	Kerrison punch	84
Gurler, et al. [3]		Drill	88.9
Wormald [6]	Australia	Drill	95.7
Kim, et al. [8]	Korea	Kerrison punch	90.5
Codere, et al. [10]	Canada	Kerrison punch	98
Graz-Cabrerizo, et al. [13]	Spain	Kerrison punch	83
Naraghi, et al. [14]	Iran	Kerrison punch	95
Agarwal [15]	India	Kerrison punch	94 (100 % with Revisions)
Yoshida, et al. [16]	Japan	Drill	93.6
Saratziotis, et al. [17]	Greece	Drill	97.8
Jin, et al. [18]	Korea	Drill	96
Razavi, et al. [20]	Iran	Kerrison punch	96
Current study	Saudi Arabia	Kerrison punch	87.88
		Drill	90.91

Operating time is a valuable factor in health care economics, ranges approximately from 750 to 2200 dollars per hour operating time in USA & Europe [19–23]. In addition, less operating time may accomplish increased surgical efficiency, volume of performed cases, and reduction of patient’s waiting list, especially in high volume setting centres [19–22]. Our results showed that there is a statistically significant difference between operating time for endoscopic DCR using the drill compared with kerrison punch. Powered drill need more time for setup, irrigation during drilling, and suctioning after that to remove generated bony dust, with meticulous use to prevent any injure to surrounding vital structures [24].

Our overall rate of minor complication (18 %) between the powered versus non powered group showed no statistical difference and was generally similar to some previous studies on endoscopic DCR [2, 8, 13]. A recent article from Germany by Horn et al. reported a minor complication rate of 10 % [25]. Rahman et al. reported a minor complication rate of 23.8 % [26]. Despite meticulous work with the drill, we observed two cases with minor nostril burn indicating a requirement of more carefulness in handling the drill [3, 27, 28].

The limitations of this study are mainly the retrospective design of the study and the moderately small sample size.

## Conclusion

No significant difference was found between the powered and the non powered groups in terms of success rate and complications. Non-powered kerrison punch showed significant reduction in operating time compared to powered drill for endoscopic DCR. Larger prospective studies are advisable before any generalization can be made.
